# Supplementary material for: Secondary attack rates of COVID-19 in Norwegian families: a nation-wide register-based study
Source: Eur J Epidemiol. 2021 May 25;36(7):741–8. doi: 10.1007/s10654-021-00760-6 (PMC8147908; doi:10.1007/s10654-021-00760-6)
Supplement: Supplementary file 1 — Supplementary material 1 (DOCX 1014 kb) [file 10654_2021_760_MOESM1_ESM.docx]

Supplementary material for the paper

Secondary attack rates of COVID-19 in Norwegian families: A nation-wide register-based study, by Kjetil Telle, Silje B. Jørgensen, Rannveig Hart, Margrethe Greve-Isdahl and Oliver Kacelnik

Supplement Figure A. Secondary PCR confirmed SARS-CoV-2 infections in all non-index family members by given number of days after the date when index family member was positive, and analogously for percent of non-index family members who were tested. All families in Norway with at least one parent and one child, where at least one family member tested positive for SARS-CoV-2 in a PCR test in 2020.


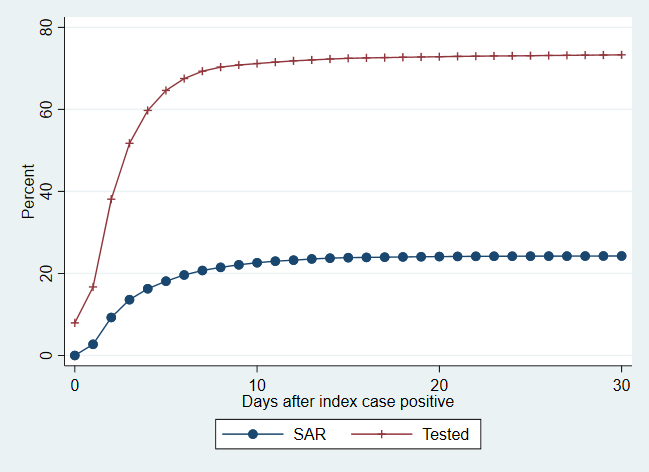


Supplement Table A. Secondary PCR confirmed SARS-CoV-2 infections in non-index family members within 14 days after index sampling date (SAR14). The data include all families in Norway consisting of at least one parent and one child, with at least one family member positive for SARS-CoV-2 in a PCR test in 2020. Percent (95% CI) and absolute numbers.

|  | Overall | | Among parents | | Among children | | Family members tested | |
| --- | --- | --- | --- | --- | --- | --- | --- | --- |
| Characteristics of the index case | SAR14 | Numerator/  Denominator | SAR14 | Numerator/  denominator | SAR14 | Numerator/  denominator | Rate | Numerator/  denominator |
| Overall | 24 (23-24) | 4613/19443 | 27 (26-28) | 1930/7219 | 22 (21-23) | 2683/12224 | 72 (72-73) | 14053/19443 |
| Parents | 28 (27-28) | 3496/12695 | 38 (36-40) | 1211/3196 | 24 (23-25) | 2285/9499 | 68 (67-69) | 8614/12695 |
| Mother | 26 (25-27) | 1910/7266 | 36 (33-38) | 552/1543 | 24 (23-25) | 1358/5723 | 68 (67-69) | 4912/7266 |
| Father | 29 (28-30) | 1586/5429 | 40 (38-42) | 659/1653 | 25 (23-26) | 927/3776 | 68 (67-69) | 3702/5429 |
| Age groups (years) |  |  |  |  |  |  |  |  |
| …. ≤30 | 30 (18-23) | 180/888 | 36 (30-43) | 70/192 | 16 (13-19) | 110/696 | 59 (55-62) | 521/888 |
| …. 31-40 | 24 (23-25) | 1129/4702 | 35 (33-38) | 387/1094 | 21 (19-22) | 742/3608 | 65 (64-66) | 3062/4702 |
| …. 41-50 | 30 (28-31) | 1526/5161 | 39 (36-42) | 507/1301 | 26 (25-28) | 1019/3860 | 72 (70-73) | 3691/5161 |
| …. ≥50 | 34 (32-36) | 661/1944 | 41 (37-45) | 247/609 | 31 (29-34) | 414/1335 | 69 (67-71) | 1340/1944 |
| Children | 17 (16-17) | 1117/6748 | 18 (17-19) | 719/4023 | 15 (13-16) | 398/2725 | 81 (80-82) | 5439/6748 |
| Daughter | 16 (15-17) | 536/3406 | 16 (15-18) | 334/2025 | 15 (13-17) | 202/1381 | 80 (79-82) | 2734/3406 |
| Son | 17 (16-19) | 581/3342 | 19 (18-21) | 385/1998 | 15 (13-17) | 196/1344 | 81 (80-82) | 2705/3342 |
| Age groups (years) |  |  |  |  |  |  |  |  |
| ….≤6 | 27 (23-31) | 130/487 | 29 (24-34) | 84/290 | 23 (18-30) | 46/197 | 92 (89-94) | 449/487 |
| …. 7-12 | 17 (15-19) | 252/1455 | 21 (19-24) | 171/803 | 12 (10-15) | 81/652 | 88 (86-89) | 1276/1455 |
| …. 13-16 | 17 (15-19) | 358/2109 | 18 (16-21) | 220/1198 | 15 (13-18) | 138/911 | 87 (86-89) | 1843/2109 |
| …. 17-20 | 14 (13-15) | 377/2697 | 14 (13-16) | 244/1732 | 14 (12-16) | 133/965 | 69 (68-71) | 1871/2697 |
| Members in family |  |  |  |  |  |  |  |  |
| 2 | 17 (15-19) | 237/1427 | 13 (10-16) | 63/484 | 18 (16-21) | 174/943 | 67 (64-69) | 954/1427 |
| 3 | 24 (23-25) | 1136/4698 | 28 (26-30) | 579/2098 | 21 (20-23) | 557/2600 | 71 (70-73) | 3347/4698 |
| 4 | 23 (22-24) | 1593/7059 | 27 (25-28) | 750/2820 | 20 (19-21) | 843/4239 | 74 (73-75) | 5193/7059 |
| 5 | 24 (23-25) | 970/4044 | 28 (26-31) | 362/1290 | 22 (21-24) | 608/2754 | 73 (72-75) | 2969/4044 |
| 6 or more | 30 (27-32) | 448/1510 | 32 (27-36) | 122/387 | 29 (26-32) | 326/1123 | 71 (69-73) | 1070/1510 |

Note: Secondary attack rate (SAR14) was calculated as the number of non-index family members who tested positive within 14 days after the date when the index family member tested positive, divided by all non-index family members, multiplied by 100. Percentage tested was calculated as the number of non-index family members who were tested within 14 days after the date when the index family member tested positive, divided by all non-index family members. 95% CIs around the estimated secondary infection rates and percentage tested were calculated using the Wilson method.

Supplement Table B. Secondary PCR confirmed SARS-CoV-2 infections in non-index family members within seven days after index sampling date (SAR7). The data include all families in Norway consisting of at least one parent and one child, with at least one family member positive for SARS-CoV-2 in a PCR test **from July 1^st^ through 2020**. Percent (95% CI) and absolute numbers.

|  | Overall | | Among parents | | Among children | | Family members tested | |
| --- | --- | --- | --- | --- | --- | --- | --- | --- |
| Characteristics of the index case | SAR7 | Numerator/  denominator | SAR7 | Numerator/  denominator | SAR7 | Numerator/  denominator | Rate | Numerator/  denominator |
| Overall | 23 (22-23) | 3673/16279 | 24 (23-25) | 1543/6323 | 21 (21-22) | 2130/9956 | 78 (78-79) | 12763/16279 |
| Parents | 28 (27-29) | 2781/9834 | 39 (37-40) | 956/2481 | 25 (24-26) | 1825/7353 | 77 (77-78) | 7608/9834 |
| ..Mother | 27 (26-29) | 1534/5607 | 37 (34-39) | 437/1195 | 25 (24-26) | 1097/4412 | 78 (77-79) | 4373/5607 |
| ..Father | 30 (28-31) | 1247/4227 | 40 (38-43) | 519/1286 | 25 (23-26) | 728/2941 | 77 (75-78) | 3235/4227 |
| Age groups (years) |  |  |  |  |  |  |  |  |
| …. ≤30 | 19 (16-22) | 139/745 | 36 (29-44) | 59/164 | 14 (11-17) | 80/581 | 63 (59-66) | 466/745 |
| …. 31-40 | 25 (24-26) | 944/3774 | 35 (32-39) | 314/888 | 22 (20-23) | 630/2886 | 72 (71-74) | 2735/3774 |
| …. 41-50 | 31 (29-32) | 1207/3922 | 40 (37-43) | 399/997 | 28 (26-29) | 808/2925 | 83 (82-85) | 3274/3922 |
| …. ≥50 | 35 (33-38) | 491/1393 | 43 (38-47) | 184/432 | 32 (29-35) | 307/961 | 81 (79-83) | 1133/1393 |
| Children | 14 (13-15) | 892/6445 | 15 (14-16) | 587/3842 | 12 (11-13) | 305/2603 | 80 (79-81) | 5155/6445 |
| ..Daughter | 13 (12-14) | 412/3243 | 14 (12-15) | 264/1926 | 11 (10-13) | 148/1317 | 80 (79-82) | 2605/3243 |
| Son | 15 (14-16) | 480/3202 | 17 (15-19) | 323/1916 | 12 (11-14) | 157/1286 | 80 (78-81) | 2550/3202 |
| Age groups (years) |  |  |  |  |  |  |  |  |
| ….≤6 | 23 (20-27) | 107/461 | 27 (22-32) | 73/273 | 18 (13-24) | 34/188 | 90 (87-93) | 417/461 |
| …. 7-12 | 14 (12-16) | 189/1367 | 18 (15-21) | 134/751 | 9 (7-11) | 55/616 | 89 (87-90) | 1214/1367 |
| …. 13-16 | 14 (13-16) | 295/2063 | 16 (14-18) | 187/1179 | 12 (10-15) | 108/884 | 86 (84-87) | 1772/2063 |
| …. 17-20 | 12 (11-13) | 301/2554 | 12 (10-13) | 193/1639 | 12 (10-14) | 108/915 | 69 (67-70) | 1752/2554 |
| Members in family |  |  |  |  |  |  |  |  |
| 2 | 16 (14-18) | 194/1197 | 12 (9-15) | 54/460 | 19 (16-22) | 140/737 | 72 (69-74) | 858/1197 |
| 3 | 24 (22-25) | 930/3930 | 26 (24-28) | 476/1830 | 22 (20-23) | 454/2100 | 78 (76-79) | 3055/3930 |
| 4 | 21 (20-22) | 1250/5838 | 24 (22-26) | 582/2430 | 20 (18-21) | 668/3408 | 80 (79-81) | 4698/5838 |
| 5 | 22 (21-23) | 766/3468 | 25 (23-28) | 291/1148 | 20 (19-22) | 475/2320 | 79 (77-80) | 2725/3468 |
| 6 or more | 28 (26-30) | 349/1250 | 29 (25-34) | 97/332 | 27 (25-30) | 252/918 | 77 (74-79) | 959/1250 |

Note: Secondary attack rate (SAR7) was calculated as the number of non-index family members who tested positive within seven days after the date when the index family member tested positive, divided by all non-index family members, multiplied by 100. Percentage tested was calculated as the number of non-index family members who were tested within seven days after the date when the index family member tested positive, divided by all non-index family members. 95% CIs around the estimated secondary infection rates and percentage tested were calculated using the Wilson method.

Supplement Table C. Secondary PCR confirmed SARS-CoV-2 infections in non-index family members within 14 days after index sampling date (SAR14). The data include all families in Norway consisting of at least one parent and one child, with at least one family member positive for SARS-CoV-2 in a PCR test **from July 1^st^ through 2020**. Percent (95% CI) and absolute numbers.

|  | Overall | | Among parents | | Among children | | Family members tested | |
| --- | --- | --- | --- | --- | --- | --- | --- | --- |
| Characteristics of the index case | SAR14 | Numerator/  denominator | SAR14 | Numerator/  denominator | SAR14 | Numerator/  denominator | Rate | Numerator/  denominator |
| Overall | 26 (25-27) | 4213/16279 | 27 (26-29) | 1732/6323 | 25 (24-26) | 2481/9956 | 81 (81-82) | 13236/16279 |
| Parents | 32 (31-33) | 3137/9834 | 42 (40-44) | 1039/2481 | 29 (28-30) | 2098/7353 | 81 (80-81) | 7920/9834 |
| Mother | 31 (29-32) | 1715/5607 | 40 (37-42) | 473/1195 | 28 (27-29) | 1242/4412 | 81 (80-82) | 4550/5607 |
| Father | 34 (32-35) | 1422/4227 | 44 (41-47) | 566/1286 | 29 (27-31) | 856/2941 | 80 (78-81) | 3370/4227 |
| Age groups (years) |  |  |  |  |  |  |  |  |
| …. ≤30 | 22 (20-26) | 167/745 | 38 (31-46) | 63/164 | 18 (15-21) | 104/581 | 66 (62-69) | 489/745 |
| …. 31-40 | 28 (26-29) | 1053/3774 | 39 (36-42) | 346/888 | 24 (23-26) | 707/2886 | 76 (75-78) | 2876/3774 |
| …. 41-50 | 35 (33-36) | 1355/3922 | 43 (40-46) | 430/997 | 32 (30-33) | 925/2925 | 86 (85-87) | 3379/3922 |
| …. ≥50 | 40 (38-43) | 562/1393 | 46 (42-51) | 200/432 | 38 (35-41) | 362/961 | 84 (82-86) | 1176/1393 |
| Children | 17 (16-18) | 1076/6445 | 18 (17-19) | 693/3842 | 15 (13-16) | 383/2603 | 82 (82-83) | 5316/6445 |
| Daughter | 16 (14-17) | 508/3243 | 16 (15-18) | 316/1926 | 15 (13-17) | 192/1317 | 82 (81-84) | 2672/3243 |
| Son | 18 (16-19) | 568/3202 | 20 (18-22) | 377/1916 | 15 (13-17) | 191/1286 | 83 (81-84) | 2644/3202 |
| Age groups (years) |  |  |  |  |  |  |  |  |
| ….≤6 | 26 (22-30) | 121/461 | 29 (24-34) | 78/273 | 23 (17-29) | 43/188 | 93 (91-95) | 430/461 |
| …. 7-12 | 18 (16-20) | 241/1367 | 22 (19-25) | 163/751 | 13 (10-16) | 78/616 | 90 (89-92) | 1235/1367 |
| …. 13-16 | 17 (15-19) | 349/2063 | 18 (16-21) | 216/1179 | 15 (13-18) | 133/884 | 88 (87-90) | 1824/2063 |
| …. 17-20 | 14 (13-16) | 365/2554 | 14 (13-16) | 236/1639 | 14 (12-17) | 129/915 | 72 (70-73) | 1827/2554 |
| Members in family |  |  |  |  |  |  |  |  |
| 2 | 18 (16-20) | 213/1197 | 12 (10-16) | 57/460 | 21 (18-24) | 156/737 | 75 (72-77) | 895/1197 |
| 3 | 27 (25-28) | 1044/3930 | 28 (26-31) | 521/1830 | 25 (23-27) | 523/2100 | 80 (79-82) | 3163/3930 |
| 4 | 25 (23-26) | 1434/5838 | 27 (26-29) | 662/2430 | 23 (21-24) | 772/3408 | 83 (82-84) | 4845/5838 |
| 5 | 26 (24-27) | 894/3468 | 29 (26-31) | 329/1148 | 24 (23-26) | 565/2320 | 81 (80-82) | 2816/3468 |
| 6 or more | 33 (31-36) | 417/1250 | 34 (29-40) | 114/332 | 33 (30-36) | 303/918 | 82 (80-84) | 1024/1250 |

Note: Secondary attack rate (SAR14) was calculated as the number of non-index family members who tested positive within 14 days after the date when the index family member tested positive, divided by all non-index family members, multiplied by 100. Percentage tested was calculated as the number of non-index family members who were tested within 14 days after the date when the index family member tested positive, divided by all non-index family members. 95% CIs around the estimated secondary infection rates and percentage tested were calculated using the Wilson method.
